# *Cetacean morbillivirus* in Northern and Southern Hemispheres

**DOI:** 10.3389/fmicb.2014.00211

**Published:** 2014-05-07

**Authors:** Giovanni Di Guardo, Sandro Mazzariol

**Affiliations:** ^1^Faculty of Veterinary Medicine, University of TeramoTeramo, Italy; ^2^Department of Comparative Biomedicine and Food Science, University of PaduaPadua, Italy

**Keywords:** *Cetacean morbillivirus*, stranded cetaceans, viral strains, virus-host interaction, aquatic mammals

In the last 25 years, at least 10 dramatic morbilliviral epidemics have occurred among free-ranging pinniped and cetacean species and populations worldwide. The origin(s) of the new *Morbillivirus* genus members causing these mass mortality events, along with the reason(s) behind their “sudden” appearance among wild aquatic mammals, are still unknown (Di Guardo et al., 2005, 2011; Di Guardo, 2012).

Interestingly enough, two recent papers have reported the existence of *Cetacean morbillivirus* (CeMV) strains infecting wild cetaceans (*Tursiops aduncus* and *Sotalia guianensis*, respectively) in the Southern Hemisphere (Groch et al., 2014; Stephens et al., 2014) and exhibiting marked genetic differences from those infecting cetaceans of the Northern Hemisphere (Van Bressem et al., 2009; Di Guardo et al., 2011; Di Guardo and Mazzariol, 2013). Albeit quantified only for the nucleoprotein (N) and phosphoprotein (P) genes, these differences are so consistent that one could question the inclusion of the concerned isolates into the CeMV clade. Furthermore, the agent recovered from the two *T. aduncus* individuals stranded along the Western Australia coastline was deemed to be, with exclusive reference to the N and P genes, the CeMV more closely related to *Measles Virus* (MeV) as well as to *Peste des Petits Ruminants Virus* (PPRV) and *Rinderpest Virus* (RPV), the archetype morbillivirus (Stephens et al., 2014). Intriguingly, no peculiar inflammatory lesions nor immunohistochemical (IHC) evidence of morbilliviral antigen were observed in the brain of these two animals (Stephens et al., 2014), differently from CeMV-infected cetaceans (Di Guardo and Mazzariol, 2013; Di Guardo et al., 2013) but also similarly to experimentally RPV-infected cattle (Wohlsein et al., 1993). In this respect, while CeMV sequences could be demonstrated in the brain and lung from approximately 40% of the striped dolphins (*Stenella coeruleoalba*) found stranded during the first mid of 2013 along the Tyrrhenian coast of Italy, IHC evidence of morbilliviral antigen was obtained from the brain of only one of these animals (Casalone et al., 2014). Nevertheless, the isolate(s) characterized from these dolphins, apart from being identical to each other, were also very similar to those responsible for the three previous epidemic outbreaks occurred throughout the last 25 years in the Western Mediterranean (Di Guardo and Mazzariol, 2013; Casalone et al., 2014).

In conclusion, while more data are certainly needed on the genetic “make-up” of the newly discovered CeMV strains infecting cetaceans in the Southern Hemisphere (Groch et al., 2014; Stephens et al., 2014), it should be additionally emphasized that, despite the existence of single, highly valuable contributions in this field (Shimizu et al., 2013), a very limited body of knowledge is available on the host-related components as well as on the agent-related factors driving the complex *Morbillivirus*-cetacean interaction dynamics (Di Guardo, 2012).

## Conflict of interest statement

The authors declare that the research was conducted in the absence of any commercial or financial relationships that could be construed as a potential conflict of interest.
